# Nonlinear Catch-Up Growth in Height, Weight, and Head Circumference from Birth to Adolescence: A Longitudinal Twin Study

**DOI:** 10.21203/rs.3.rs-2005347/v1

**Published:** 2023-02-10

**Authors:** Sean R. Womack, Christopher R. Beam, Evan J. Giangrande, Rebecca J. Scharf, Xin Tong, Medha Ponnapalli, Deborah W. Davis, Eric Turkheimer

**Affiliations:** University of Virginia; University of Southern California; University of Virginia; University of Virginia Medical Center; University of Virginia; University of Virginia; University of Louisville; University of Virginia

**Keywords:** Height, weight, head circumference, catch-up growth, twin

## Abstract

Owing to high rates of prenatal complications, twins are, on average, substantially smaller than population norms on physical measurements including height, weight, and head circumference at birth. By early childhood, twins are physically average. This study is the first to explore the process of catch-up growth by fitting asymptotic growth models to age-standardized height, weight, and head circumference measurements in a community sample of twins (*n* = 1,281, 52.3% female) followed at up to 17 time points from birth to 15 years. Catch-up growth was rapid over the first year and plateaued around the population mean by early childhood. Shared environmental factors accounted for the majority of individual differences in initial physical size (57.7%−65.5%), whereas additive genetic factors accounted for the majority of individual differences in the upper asymptotes of height, weight, and head circumference (73.4%−92.6%). Both additive genetic and shared environmental factors were associated with variance in how quickly twins caught up. Gestational age and family SES emerged as important environmental correlates of physical catch-up growth.

## Introduction

As early as 24 weeks gestation, twins display a dampened rate of physical growth relative to singletons (Hiersch et al., 2020). Discrepancies in prenatal growth rates may be due to uterine size constraints (Blickstein, 2004), increased nutrition requirements of two fetuses (Liao et al., 2012), or elevated rates of maternal health complications in twin pregnancies (Buhling et al., 2003; Rauh-Hain et al., 2008). Relative to singletons, twins are 7.2 times more likely to be born prematurely and 8.3 times more likely to be born at low birth weight (Martin et al., 2021). In infancy, community samples of twins are approximately a standard deviation smaller than their singleton peers on measures of physical development including height, weight, and head circumference (Estourgie-van Burk et al., 2010; van Dommelen et al., 2008; Wilson, 1974; Wilson, 1979). However, by early childhood, twins are average in terms of height, weight, and head circumference (Sundet et al., 2005; Wilson, 1979). Thus, between birth and early childhood, the average twin displays considerable physical catch-up growth. This study is the first to explore the process of catch-up growth in twins by fitting latent growth models to standardized measurements of height, weight, and head circumference in the Louisville Twin Study (LTS), a community sample of twins followed from birth to adolescence (Beam et al., 2021). Leveraging the genetically informative portion of the twin study, we also explored additive genetic, shared environmental, and nonshared environmental factors associated with individual differences in trajectories of height, weight, and head circumference catch-up.

Deficits in physical size at birth are linked to a myriad of negative developmental outcomes including delays in early cognitive and motor development (Kuklina et al., 2006; Scharf et al., 2016; Upadhyay et al., 2019). Sustained Deficits in physical size increase the risk for poor neurodevelopment and other serious health concerns across the lifespan. For example, length, weight, and head circumference measurements greater than 2 SD below the population mean in infancy are associated with an increased risk of receiving a diagnosis of global developmental delay or intellectual disability (Bélanger & Carone, 2015). Likewise, being clinically underweight in adulthood (BMI < 18.5 kg/m^2^) is a risk factor for all-cause mortality (Flegal et al., 2005). Therefore, identifying the typical rate and shape of catch-up growth in height, weight, and head circumference is important to identify children that are at risk for abnormal physical development and subsequent neurodevelopmental and health outcomes. Likewise, identifying environmental correlates of catch-up growth in children exposed to early bio-environmental adversity is a first step in informing interventions to promote healthy physical development. Finally, as twins can serve as a developmental model for singletons, understanding the process of catch-up growth in twins may more broadly inform our understanding of singleton development following exposure to early bio-environmental stressors (e.g., prematurity, gestational diabetes).

### Catch-up Growth In Height, Weight, And Head Circumference

Among singletons born small for gestational age, most catch-up growth in height occurs in the first 12 months, suggesting that catch-up growth of physical size may begin in infancy (Brandt et al., 2003; Itabashi et al., 2007). Accordingly, twins make rapid physical gains toward singleton norms across infancy, but Deficits in height, weight, and head circumference remain apparent in toddlerhood (Buckler & Green, 2004; van Dommelen et al., 2008). By late childhood, most samples of twins suggest there are minimal to no discrepancies in physical size between twins and singletons (Estourgie-van Burk et al., 2010; Wilson, 1979). For example, at birth, twins in the LTS were 30% lighter and 17% shorter than singleton norms (Wilson, 1979). However, twins in the LTS were within 5% of singleton norms by 5 years and exhibited no differences in height or weight by 8 years (Wilson, 1979). Although Wilson did not fit growth models to the physical growth data, plots of height and weight means over time appear exponential with rapid catch-up occurring in the first months and leveling off around the singleton means by early childhood. Community twin samples have average heights, weights, and head circumferences relative to population norms in adolescence and adulthood, indicating that early physical catch-up is maintained at later developmental stages (Estourgie-van Burk et al., 2010; Sundet et al., 2005; Wilson, 1979). Thus, the typical pattern of catch-up growth appears to be asymptotic with gains in height and weight stabilizing around the population mean in middle childhood and sustaining into adulthood.

### Biometric Analyses Of Height, Weight, And Head Circumference

Biometric twin studies that decompose the variance of a phenotype into genetic and environmental components generally suggests that additive genetic factors account for an increasing proportion of the variance in height, weight, and head circumference across the lifespan (Estourgie-van Burk et al., 2010; Mook-Kanamori et al., 2012; Smit et al., 2010). The heritability of birth weight, length, and head circumference tends to be relatively low (Mook-Kanamori et al., 2012; Smit et al., 2010), which may reflect strong associations between prenatal environmental experiences and individual differences in early physical size. For example, analysis of four samples of twins from the Netherlands and Australia found the shared environment to contribute to between 71% and 82% of the variance in head circumference among infants younger than four months and additive genetics to contribute to between 84% and 92% of the variance in head circumference for infants between 5 and 13 months (Smit et al., 2010). Likewise, Mook-Kanamori and colleagues (2012) found additive genetics to account for approximately 27% of the variance in birth length and 29% of the variance in birth weight, whereas the shared environment accounted for 46% and 22% of the variance in birth length and weight, respectively. By 36 months, additive genetics accounted for 72% of the variance in height and 71% of the variance in weight and the shared environment accounted for 17% and 14% of the respective variance in height and weight at 36 months (Mook-Kanamori et al., 2012). Studies of adult samples have found height and weight to be highly heritable with heritability estimates for height, weight, and head circumference ranging from .68 to .90 (Silventoinen et al., 2000; Silventoinen et al., 2003; Smit et al., 2010).

Although there are numerous studies that have conducted biometric analyses on height and weight in twins at specific developmental periods, biometric analyses of the change in physical size over time are relatively uncommon. Moreover, previous biometric analyses of the change in physical size have primarily focused on a brief developmental window of infancy and early toddlerhood, limiting our understanding of genetic and environmental contributions to individual differences in physical development across childhood. For example, Demerath and colleagues (2007) found additive genetic factors to account for 82% of the variance in the magnitude of change in weight-for-age z-scores between birth and 24 months. However, this sample was restricted to twins born at full term and only measured weight at two occasions, prohibiting the exploration of biometric contributions to the rate of change. On the other hand, Livshits and colleagues (2000) found shared environmental factors to account for the majority of the variance in the velocity of height, weight, and head circumference growth over the first 12 months. Additive genetics did not account for a significant proportion of the variance of velocity of height or weight growth (Livshits et al., 2000). Research extending into toddlerhood and early childhood has found that a combination of additive genetic and shared environmental factors are related to individual differences in trajectories of height and weight development (Johnson et al., 2011; van Dommelen et al., 2004).

### Specific Environmental Contributions To Catch-up Growth

Exposure to prenatal stressors reflects one aspect of the shared environment that may contribute to early physical development. Gestational age represents a crude, but useful, indicator of exposure to prenatal insults (Kline et al., 1989). Among a sample of Israeli twins, length of gestation accounted for approximately 53% of the variance in birth weight, 41% of the variance in birth length, and 51% of the variance in head circumference at birth (Livshits et al., 2000). However, the rate of height, weight, and head circumference growth over the first year was unrelated to gestational age (Livshits et al., 2000). In a sample of Dutch twins, gestational age was positively associated with height at birth, and twins born at a greater gestational age had a steeper deceleration in the rate of height growth between birth and 24 months (van Dommelen et al., 2004).

Socioeconomic status, which may reflect access to higher quality nutrition and medical care, is an aspect of the postnatal environment that has been associated with physical development (Ashworth et al., 1997; Batista et al., 2012; Jelenkovic et al., 2020). Among Brazilian singletons born at low birthweight, family SES explained 21.4% of the variance in catch-up weight gain over the first year and 24.4% of the variance in catch-up height, accounting for maternal height, maternal smoking, and prenatal illness (Ashworth et al., 1997). Results from a large aggregation of twin samples from 15 countries revealed a modest, positive correlation between height and parental education (Jelenkovic et al., 2020). In Western cultures, some research suggests an inverse relationship between SES and early weight gain such that infants in poorer households gain weight more rapidly than infants in wealthier families (Wijlaars et al., 2011). Thus, in the process of catching up to the population mean in terms of weight, twins in poorer homes may demonstrate a steeper rate of weight gain and ultimately overshoot the population mean, ending up overweight in early childhood.

### The Present Study

The present study seeks to describe the process by which twins catch-up to the population mean in terms of height, weight, and head circumference. Specifically, we fit nonlinear growth models to height for age, weight for age, and head circumference for age z-scores from birth to 15 years (birth to three years for head circumference) to model the magnitude, rate, and shape of catch-up growth of physical size in twins. We then conducted biometric analyses on the best fitting growth models to determine relative additive genetic, shared environmental, and nonshared environmental contributions to catch-up growth. Finally, we examined measured prenatal and postnatal correlates to trajectories of catch-up growth across childhood. The study design and hypotheses were preregistered and all R and Mplus scripts are available on the Open Science Framework (https://osf.io/dsyac/).

## Methods

### Participants

Participants were 1,281 individual twins recruited as a part of the LTS, an ongoing longitudinal study of temperament and intellectual development in twins (Davis et al., 2019). All twins were recruited from the Louisville, Kentucky metropolitan area. Participants were primarily White (91.5%) and were recruited to represent the socioeconomic composition of the Louisville metropolitan area at participant enrollment. Twin zygosity was determined by blood serum analysis. Over the 36-year course of the LTS, there were 1,770 individuals (885 pairs) listed as participating at least once. Twins missing zygosity information will not be included in analyses (*n* = 120). Of those remaining, 1,637 had at least one physical measurement. As is typical in twin studies, we restricted analyses to monozygotic and same-sex dizygotic twins (*n* = 1,287). Finally, provided my focus on typical physical development, we removed all individuals with height, weight, or head circumference measurements greater than 4 SD above or below the population mean (*n* = 6). Thus, the final sample is 1,281 (52.3% female).

### Procedure

Data were collected between 1957 and 1993. Physical measurements were collected by trained examiners during laboratory visits at the University of Louisville at 16 time points between 3 months and 15 years (0.25, 0.5, 0.75, 1, 1.5, 2, 2.5, 3, 4, 5, 6, 7, 8, 9, 12, and 15 years). Most individuals in the study did not have data at all 16 points, but 88.2% had four or more weight measurements, 84.7% had four or more height measurements, and 87.3% had four or more head circumference measurements. All study procedures were approved by the University of Louisville Institutional Review Board. Informed consent was obtained from all participants included in this study.

### Measures

#### Physical development.

Physical measurement procedures are described in detail elsewhere (Wilson, 1974, Wilson, 1979). Birth length, weight, and head circumference measurements were obtained from birth certificates. All subsequent physical measurements were taken during assessments conducted at the University of Louisville. Infant weights between 3 and 24 months were taken with the infant lying undressed on a balance scale. After 24 months, infants were weighed wearing a light garment using a platform scale calibrated in four-ounce increments. All weights were recorded to the nearest ounce and were subsequently converted to kilograms. Height was measured to the nearest millimeter. Recumbent length was use as a proxy for height between 3 and 24 months. After 24 months, standing height was measured using a wall-mounted metric scale. Head circumference was measured to the nearest eighth of an inch and converted to metric measurements for analyses (Wilson, 1974). Raw height, weight, and head circumference measurements were converted into age-standardized z-scores using Center for Disease Control (CDC) growth charts based on 2000 norms (Kuczmarski, 2000). Z-scores are calculated separately for males and females and, therefore, are also standardized by sex. The 2000 CDC growth charts were based on United States population surveys conducted between 1963 and 1994, which overlaps with the timeline of data collection in the LTS.

#### Covariates.

Baseline household SES was measured based on the Hollingshead Four Factor Index of Socioeconomic Status, which is based on parental occupation, education, sex, and income (Hollingshead, 1975). Hollingshead scores are based on a continuous zero to 100-point scale. Gestational age (in weeks) was calculated based on maternal report of last menses. Maternal age (in years) at birth and child sex were also included as covariates in growth models. Continuous covariates were standardized to have a mean of 0 and SD of 1, and sex was centered. A quadratic SES term was created by squaring the standardized SES term. We centered the quadratic SES term to ensure it had a mean of 0.

### Data Analysis

#### Nonlinear Growth Modeling.

We considered a variety of growth models to describe typical trajectories of height and head circumference including polynomial (linear, quadratic, and cubic), exponential, and sigmoid-shaped (Logistic, Gompertz, Richard’s, Weibull, and Morgan-Fercer-Flodin) growth models. Because models were not nested, the best fitting model was determined by comparing Bayesian Information Criterion (BIC) values across models with lower values indicating better overall model fit. We review the characteristics of the Weibull and Morgan-Mercer-Flodin growth models as these models fit the physical growth data best.

Weibull Growth Model. The Weibull growth model is a nonlinear, asymptotic curve derived from the Weibull distribution (Ratkowsky, 1983). Using this model, the predicted height for individual we at time *t* is estimated using the following equation.

$${\text{HEIGHT}}_{\text{it}}={\text{b}}_{1\text{i}}-\left({\text{b}}_{1\text{i}}-{\text{b}}_{0\text{i}}\right)•\text{exp}\left(-{\text{b}}_{2\text{i}}•{\text{t}}^{{\text{b}}_{\text{3i}}}\right)+{\text{e}}_{it}$$

In this model, *b*_*0*_ corresponds to the intercept, or estimated weight when time equals zero (i.e., birth weight). *b*_*1*_ corresponds to the upper asymptote, or an individual’s maximum estimated weight. *b*_*2*_ reflects the average rate at which an individual approaches their upper asymptote. Finally, *b*_*3*_ is a shaping parameter that controls the inflection point of the curve or the point at which growth is the fastest. In cases where *b*_*3*_ is less than 1, the Weibull curve approximates an exponential shape, and when *b*_*3*_ is greater than 1, the Weibull curve takes a sigmoid shape. Using the *b*_*3*_ parameter, the age of most rapid growth can be calculated using the following equation:

$$\text{AgeofInflection}=\left(\frac{1}{b_{2}}\right)*{\left(\frac{b_{3}-1}{b_{3}}\right)}^{\left(\frac{1}{b_{3}}\right)}$$

In cases where the inflection scaling parameter is less than 1, the age of inflection is not defined as it is negative in cases where 1 $\left(\frac{1}{b_{3}}\right)$ is odd and not a real number when $\left(\frac{1}{b_{3}}\right)$ 1 is even.

Morgan-Mercer-Flodin Model. Using a Morgan-Mercer-Flodin model, the predicted head circumference for individual we at time *t* can be estimated using the following equation (Morgan et al., 1975).

$$HEAD_{ij}=b_{1i}-\frac{b_{1i}-b_{0i}}{\left(1+{\left(b_{2}•\text{t}\right)}^{b_{3i}}\right)}+{\text{e}}_{it}$$

In the Morgan-Mercer-Flodin model, *b*_*0*_ corresponds to the intercept, *b*_*1*_ is the upper asymptote, *b*_*2*_ is the average rate of growth. The inflection point scaling parameter (*b*_*3*_) can be used to calculate the age at which growth is the fastest using the following equation:

$$\text{AgeofInflection}={\left(\frac{b_{3}-1}{b_{3}+1}\right)}^{\frac{1}{b_{3}}}$$

Nonlinear growth functions were estimated in a structural equation modeling framework using a Taylor Series expansion to generate a linear function of the target function (Grimm et al., 2013). Specifically, this is done by fixing the factor loadings to the partial derivative of each parameter in the target function (i.e., *b*_*0*_ – *b*_*3*_). All growth models were fit using Mplus version 8.4 (Muthén & Muthén, 2017).

#### Genetic analyses.

A multilevel approach (individual twins nested within families) was used to decompose the variance estimates of the growth parameters into additive genetic, shared environmental, and nonshared environmental components (McArdle & Prescott, 2005). Following a classical twin model, additive genetic (A), shared environmental (C), and nonshared environmental (E) contributions to individual differences in the growth parameters can be derived using the following equations.

$${\text{MZ}}_{\text{within}}=\text{E}$$

$${\text{MZ}}_{\text{between}}=\text{A}+\text{C}$$

$${\text{DZ}}_{\text{within}}=0.5^{*}\text{A}+\text{E}$$

$${\text{DZ}}_{\text{between}}=0.5^{*}\text{A}+\text{C}$$

#### Environmental correlates of height and head circumference catch-up.

Constrained growth models were fit by regressing the height, weight, and head circumference growth parameters onto the study covariates. Because all study covariates were consistent across twin pair, the covariates were included at the between-pair level of the model.

### Missing Data

Due to the longitudinal nature of the study, there were missing physical growth measurements at each age (see Supplementary Table I). Missing data were handled using full information maximum likelihood estimation (FIML) in Mplus. FIML assumes that data are missing at random (MAR). When data are MAR, missingness may be related to other observed variables (e.g., family SES), but not to the missing value itself (e.g., extremely short children are more likely to have missing heights). To explore patterns of missingness, we fit a series of logistic regression models using study covariates, birth year, zygosity, and observed physical measurements at the previous age to predict the likelihood of missing height or head circumference measurements at a given age. As height, weight, and head circumference measurements across childhood are relatively stable over time (see Supplementary Figs. 1–3, respectively), using the previous measurement to predict missingness at the subsequent assessment (e.g., height at 3 months predicting missing height at 6 months) allowed us to approximate if missingness was related to the missing value. There was evidence that height and head circumference measurements were MAR (see supplementary Tables 2–4). Missingness did not appear consistently biased by smaller or larger children and birth year emerged as a consistent significant predictor of missing height, weight, and head circumference measurements. In general, children born later were more likely to be missing all physical growth measurements, which may reflect a change in the focus of the LTS from physical development in the 1950s to cognitive development in later decades or a loss of study funding in the 1990s (see supplementary Fig. 4). Birth year was included in all models as a predictor of measured height and head circumference at each age to avoid generating biased parameter estimates (Enders, 2013).

### Descriptive Statistics And Intercorrelations

Means and standard deviations of height, weight, head circumference, and study covariates are presented in Supplementary Tables 5–8, respectively. The LTS sample was of average SES, and family SES was approximately evenly distributed across quintiles (22.0%, 20.2%, 19.0%, 25.9%, and 12.8% in the first through fifth quintiles, respectively). The average length of gestation was 37.2 weeks (*SD* = 2.6 weeks; mediation gestation = 38 weeks). About a third of the infants (33.6%) were born prematurely (less than 37 weeks gestation) and 3.4% were born very prematurely (less than 32 weeks gestation). At birth, the average twin was 1.59 *SD* lighter, 0.78 *SD* shorter, and had head circumference measurements that were 1.35 *SD* below the population mean. By 15 years, the average height-for-age z-score was 0.03 (*SD* = 0.98) and the average weight-for-age z-score was 0.19 (*SD* = 0.95). Average head circumference z-scores at 36 months (the last head circumference measurement) were 0.43 *(0.89)*. Sequential height, weight, and head circumference measurements were highly correlated, suggesting strong stability of physical measurements over time (*r*’s .50–.99; see Supplementary Figs. 1–3 for height, weight, and head circumference intercorrelations across study waves, respectively).

### Height Catch-up

#### Model fit – height.

**Figure 1:**
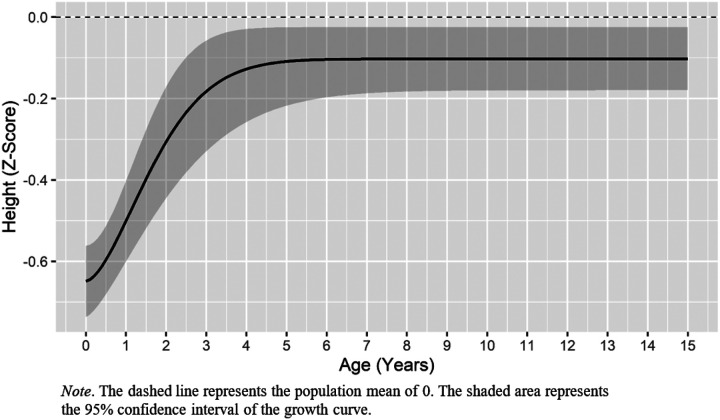
Predicted Height Catch-Up Based on the Weibull Function

Model fit statistics for the height catch-up curves are presented in Supplementary Table IX. Based on BIC values, the Weibull curve fit the data better than other growth models. Moreover, relative fit indices suggested that the Weibull curve fit the data acceptably (RMSEA = 0.08, TLI = 0.92). The estimated intercept of −0.66 indicates that the average twin was approximately two thirds of a standard deviation below the population mean in terms of birth length. The upper asymptote was estimated to be −0.11, indicating that the average twin caught up to within approximately a tenth of a standard deviation of the population mean. The inflection point was calculated to be 1.75 (95% C.I. 1.54, 1.84), indicating that height catch-up was most rapid in early toddlerhood. Estimates for all growth parameters are presented in Table I and Fig. 1 depicts the average trajectory of height catch-up.

#### Biometric analyses – height.

Biometric contributions to individual differences in height catch-up are presented in Table I. Shared environmental factors accounted for 57.7% of the variance in the intercept of height whereas additive genetic and nonshared environmental factors accounted for 28.3% and 13.9% of the variance, respectively. Additive genetic factors accounted for the majority of individual differences in the upper asymptote of height (81.4% of the variance). Individual differences in the rate of height catch-up were associated with a combination of additive genetic (37.0% of the variance) and shared environmental factors (52.5% of the variance). Shared environmental factors accounted for the majority of individual *Predicted Height Catch-Up Based on the Weibull Function*

#### Environmental correlates – height.

**Figure 2:**
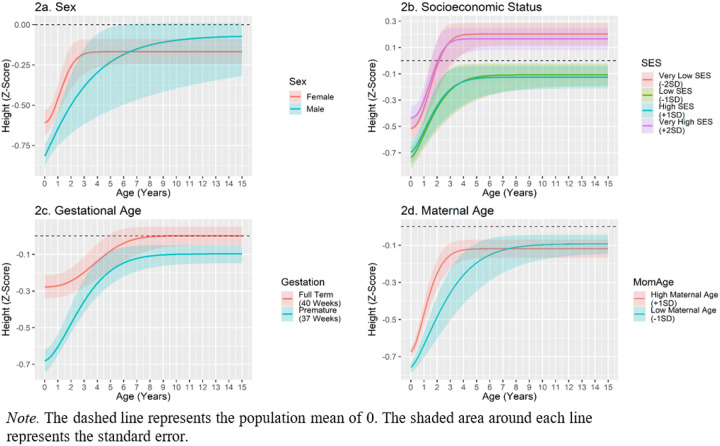
Environmental Correlates of Height Catch-Up Growth

Relative to females, males had lower initial lengths, had a slower average rate of growth, and demonstrated an earlier inflection point. Thus, males started off farther behind than females and caught up by growing slower and for a longer period of time (see Fig. 2a). Importantly, sex was not significantly related to the upper asymptote of height, indicating that males made up their initial height de cit relative to females. Family SES was not linearly related to any of the height catch-up parameters. There was a significant quadratic association between SES and the upper asymptote of height. The quadratic association suggests that SES is most strongly related to the upper asymptote of height at the extremes. Accordingly, children in the poorest homes (−2 SD) and wealthiest homes (+ 2 SD) had the highest upper asymptotes (see Fig. 2b). Gestational age was strongly associated with the intercept of height (i.e., birth length). Moreover, a longer gestation was associated with a slower rate of growth and a later inflection point. Gestational age was not significantly associated with the upper asymptote of height. Therefore, children born prematurely had substantial early height Deficits, but caught up both to their full-term peers and the population mean by growing faster (see Fig. 2c). Maternal age was positively associated with the intercept of height, but was not significantly associated with any other growth parameters (see Fig. 2d). All associations between study covariates and height catch-up are presented in Table II.

As an approximation of effect size, we calculated the proportion of the shared environmental variance explained by measured shared environmental constructs (i.e., family SES, gestational age, etc.). This was calculated by subtracting the unstandardized shared environment variance in the constrained model from the shared environmental variance in the unconstrained model and dividing the difference by the shared environmental variance in the unconstrained model (Singer et al., 2003). Study covariates accounted for24.0% of the shared environmental variance for the intercept, 23.6% of the variance in the upper asymptote, 4.0% of *Environmental Correlates of Height Catch-Up Growth*

### Weight Catch-up

#### Model fit – weight.

Model fit information for the growth curve models fit to weight can be found in Supplementary Table X. Based on the BIC values, the Weibull growth model fit the data best. Although the Weibull model fit the data best relative to the other growth models, the RMSEA value (.10) and TLI value (.88) indicated that the Weibull curve did not fit the data well. Growth curve models were initially fit following a traditional approach to modeling the residual structure: residual variances were freely estimated and covariances between residuals were omitted. Modeling the structure of the residuals by including different variance constraints or autocorrelations can improve model fit (Grimm & Widaman, 2010). There are several approaches to modeling the covariance structure of the residuals in the growth curve model, and it is generally recommended to select a structure that balances parsimony and model fit (Wolfinger, 1993). We used a banded structure to model the residual structure of the Weibull growth model fit to weight (Wolfinger, 1993; see below for a simplified covariance matrix). Using a banded structure, residual variances for each measured variable were freely estimated and covariances between sequential residuals (e.g., between 3 and 6 months) were freely estimated. However, all other residuals remained uncorrelated (e.g., 3 months and 15 years).

$$\left[\begin{array}{cccc} \sigma_{1}^{2} & \sigma_{5} & 0 & 0\\ \sigma_{5} & \sigma_{2}^{2} & \sigma_{6} & 0\\ 0 & \sigma_{6} & \sigma_{3}^{2} & \sigma_{7}\\ 0 & 0 & \sigma_{7} & \sigma_{4}^{2} \end{array}\right]$$

**Figure 3:**
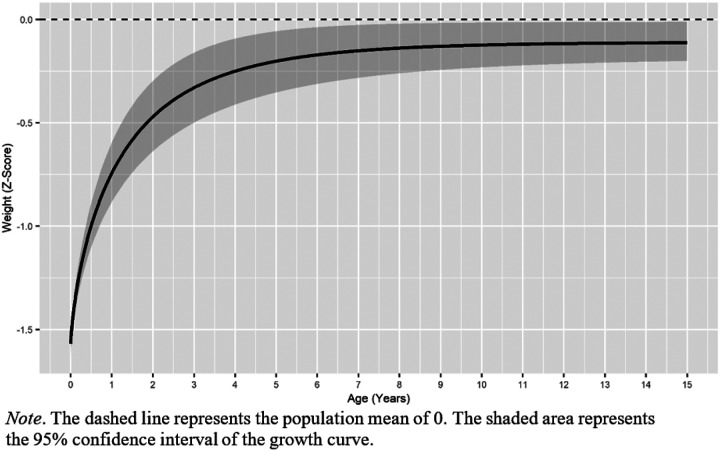
Predicted Weight Catch-Up Based on the Weibull Function

A Satorra-Bentler chi-square difference test (Satorra, 2000) revealed that the model including structured autocorrelations between the residuals fit significantly better than the model without (*X*^2^ = 815.94, df = 17, *p* < .001). The final Weibull growth model fit the data acceptably (*X*^2^ = 992.07, df = 124, *p* < .001; RMSEA = .07; TLI = .93). Parameter estimates for the final Weibull model are presented in Table III. The average twin had an intercept of −1.57, indicating that at birth the average twin was in the 6th percentile for weight. The upper asymptote was − 0.11, indicating that the average twin caught up to within about a tenth of a standard deviation of the population mean (see Fig. 3). As the scaling parameter was estimated to be less than 1, we were unable to calculate the age of inflection.

#### Biometric analyses – weight.

Unstandardized and standardized biometric components are presented in Table III. Shared environmental factors contributed to the majority of the variance in the intercept (65.5%) and did not contribute significantly to the upper asymptote. On the other hand, additive genetics did not contribute significantly to the intercept of weight, but accounted for most of the variance in the upper asymptote (73.4%). Additive genetics and shared environmental factors accounted for a significant portion of the variance in the rate of growth (67.1% and 27.3% of the variance, respectively) and inflection point (53.2% and 39.4% of the variance, respectively).

#### Environmental correlates – weight.

**Figure 4:**
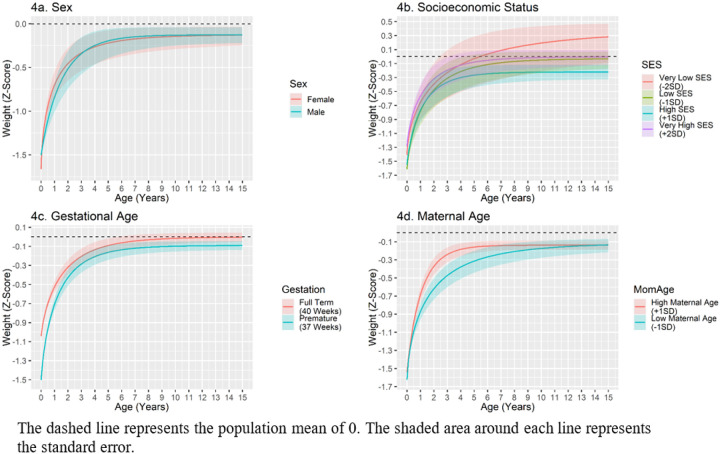
Environmental Correlates of Weight Catch-Up Growth

Males had a significantly higher intercept than females, suggesting that male twins were born closer to the population mean. However, males and females were statistically indistinguishable at the upper asymptote. Females had a faster rate of catch-up growth and an earlier inflection point than males (see Fig. 4a). The quadratic, but not the linear SES term was associated with the intercept and upper asymptote of weight catch-up. At birth, children in very low (−2 SDs) or very high (+ 2 SDs) SES homes were born the heaviest (Supplementary Fig. 5 depicts weight trajectories over the first year). By adolescence, children at very low SES had the highest weights and were continuing to grow towards an upper asymptote above the population mean whereas children in very high SES homes were approximately average (see Fig. 4b). Gestational age was positively associated with the intercept and upper asymptote of weight. Twins born at an earlier gestational age displayed a quicker rate of growth, which resulted in a narrowing of the weight gap between premature and full-term twins (see Fig. 4c). However, full-terms twins retained a slight weight advantage at the upper asymptote. Maternal age was positively associated with the inflection point of weight catch-up growth; children born to older mothers had a later inflection point (see Fig. 4d). See Table IV for all associations between covariates and weight catch-up growth.

Study covariates accounted for 54.6% of the shared environmental variance in the intercept, 24.7% of the variance in the upper asymptote, 14.7% of the variance in the rate of growth, and 6.7% of the variance in the inflection point.

### Head Circumference Catch-up

#### Model fit – head circumference.

**Figure 5:**
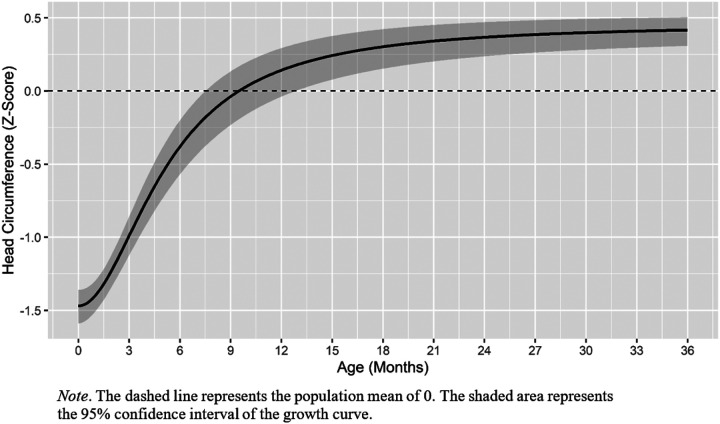
Predicted Head Circumference Catch-Up Based on the Morgan-Mercer-Flodin Function

Fit statistics for the growth curves fit to the head circumference data are presented in Supplementary Table XI. The Morgan-Mercer-Flodin growth curve fit the data best based on BIC values and fit the data well (RMSEA = .07, TLI = .96). The average twin had a intercept of −1.53 and grew to an upper asymptote of 0.40 (see Table V for all parameter estimates). The age of inflection was calculated to be 0.63. Therefore, the average twin is growing most rapidly at approximately 7.5 months of age. The average twin reached the population mean (i.e., had an estimated z-score of 0) by 9.5 months (95% C.I. 7.6, 12.9 months). Figure 5 depicts the average head circumference catch-up trajectory.

#### Biometric analyses – head circumference.

Individual differences in head circumference at birth were significantly related to shared environmental (59.4% of the variance) and nonshared environmental factors (24.0% of the variance), but were not significantly related to additive genetic factors. As with height, additive genetic factors accounted for the majority of the variance in the upper asymptote of head circumference (92.6% of the variance). Additive genetic factors also accounted for the majority of the variance in the rate of head circumference catch-up (69.3% of the variance). Additive genetic factors associated with individual differences in the inflection point of head circumference catch-up were constrained to be 0 as this parameter was initially estimated to be (non-significantly) negative. Individual differences in the inflection point of head circumference catch-up were primarily associated with shared environmental factors (65.1% of the variance). See Table V for the biometric factors associated with head circumference catch-up.

#### Environmental correlates – head circumference.

**Figure 6:**
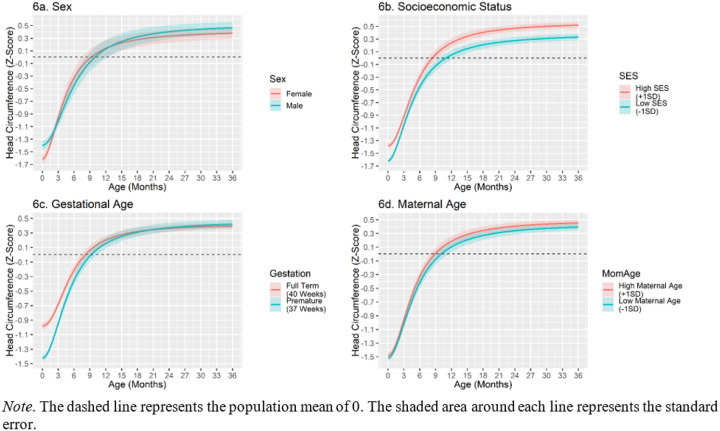
Environmental Correlates of Head Circumference Catch-Up Growth

Relative to females, males had higher head circumference measurements at birth. However, sex was not significantly related to the upper asymptote of head circumference. Females had a significantly faster rate of head circumference growth than males (see Fig. 6a for head circumference trajectories by sex). SES was linearly related to the lower and upper asymptotes. Specifically, individuals a standard deviation above the mean in SES had a 0.12 standard deviation advantage in their head circumference in infancy, and a 0.09 standard deviation advantage in the upper asymptote. Thus, the early advantages of SES associated with head circumference appear to be retained into early childhood (see Fig. 6b). Gestational age was strongly associated with the intercept of head circumference; a standard deviation increase in gestational age is associated with about a half standard deviation increase in head circumference in infancy. However, gestational age is not associated with the upper asymptote. Children born earlier had a slower rate of catch-up growth, but an earlier inflection point suggestive of a longer period of catch-up growth (see Fig. 6c). Maternal age was not significantly associated with any of the head circumference growth parameters (see Fig. 6d). See Table VI for associations between covariates and head circumference catch-up.

Study covariates accounted for 66.1% of the shared environmental variance in the intercept, 83.3% of the shared environmental variance in the upper asymptote, and 14.4% of the shared environmental variance in the inflection point. Shared environmental contributions to the upper asymptote of head circumference were nonsignificant and estimates were extremely small. Thus, the percent of variance in the upper asymptote of head circumference accounted for by study covariates should be interpreted with caution. *Environmental Correlates of Head Circumference Catch-Up Growth*

## Discussion

Nonlinear growth models fit to age-standardized measurements of height, weight, and head circumference from birth to adolescence revealed a pattern of development characterized by large Deficits in physical size at birth and rapid physical catch-up growth across infancy. Deficits in height, weight, and head circumference were comparable to observations in previous studies (Johnson et al., 2011; van Dommelen et al., 2008). Although the upper asymptotes for height and weight were statistically different from 0, the average twin was within 0.1 SD of the mean at their upper asymptote, suggesting that the difference is not clinically meaningful. Additionally, the upper asymptote for head circumference was slightly above the population mean. Thus, on average twins fully caught up to age-typical heights, weights, and head circumference, which is consistent with findings from previous research (Estourgie-van Burk et al., 2010; Wilson, 1974, Wilson, 1979).

The rate of catch-up growth for each growth metric was most rapid in infancy, highlighting the importance of catch-up growth over the first few months of life. For example, the rate of head circumference catch-up growth was most rapid at approximately 7.5 months and, on average, twins had recovered half of their initial weight Deficits by 12 months. Therefore, children born with significant Deficits in their physical size would be expected to make rapid progress toward the population mean across the first year of life. Children who do not make progress toward the population mean over the first year of life may be at an increased risk for severe health problems related to physical size including stunting, wasting, microcephalia, or failure to thrive. Temporally, twins reached their upper asymptote of head circumference first (see Supplementary Fig. 6), suggesting that neurological catch-up begins rapidly following birth and precedes catch-up growth of other structures (i.e., bone structure, adipose) in infancy.

### Biometric Contributions To Catch-up Growth

Biometric analyses of height, weight, and head circumference catch-up growth revealed a consistent pattern across measurement. Aspects of the shared environment accounted for the majority of the variance in the intercept of physical size, additive genetics accounted for the majority of the variance in the upper asymptote, and the rate and shape of catch-up growth was associated with a combination of additive genetic and shared environmental factors. The large association of shared environmental factors and nonsignificant association of genetic factors with variance in physical size at birth suggests that exposure to early bioenvironmental stress (e.g., premature birth) may override genetic contributions to early physical size. Interestingly, nonshared environmental factors explained a small but statistically significant portion of the variance in the intercepts of height, weight, and head circumference. Significant nonshared environmental contributions to early physical size may reflect differences in placental placement which lead to better nutritional access for one twin (Marceau et al., 2016) or in more extreme cases, twin-twin transfusion syndrome (Simpson et al., 2013).

On the other hand, that additive genetics accounted for the majority of the variance in the upper asymptotes of height, weight, and head circumference whereas shared environmental factors did not account for a significant portion of the variance. As children age and early environmental stressors (e.g., perinatal stressors) become more distal experiences, genetic influences on physical size may have more room to operate. Findings that additive genetics contribute to the majority of the variance in the upper asymptote of physical size are consistent with previous research that has found physical size to be highly heritable in samples above the age of 3 years (Silventoinen et al., 2000; Silventoinen et al., 2003; Smit et al., 2010). The nonsignificant contribution of shared environmental factors suggests that environmental differences between families (e.g., between-family differences in the types of food available at home) do not significantly contribute to differences in the upper asymptote of height, weight, or head circumference. However, the significant nonshared environmental contributions to the variance of the upper asymptote of physical size suggest that differences within families (e.g., within-pair differences in diet) contribute to the variance in physical size.

The present study made unique contributions to the literature by demonstrating that a combination of additive genetic, shared environmental, and nonshared environmental factors are associated with individual differences in the latent rate and shape of physical catch-up from early bioenvironmental adversity. Therefore, a combination of genetic factors (e.g., genes influencing the accumulation of body mass and bone growth) and shared environmental factors (e.g., feeding schedule, postnatal diet) are related to how quickly infant twins catch-up physically.

### Patterns Of Catch-up Growth By Sex

The intercepts of weight and head circumference were higher for males than females, suggesting that males may be more resilient to the perinatal stressors associated with twinning. However, females demonstrated a faster rate of growth of height, weight, and head circumference than males. The upper asymptotes for height, weight, and head circumference were statistically indistinguishable between males and females. Therefore, early discrepancies in relative physical size between males and females do not persist into childhood, and early discrepancies are overcome by a faster rate of catch-up growth among females. A similar pattern of height and weight catch-up growth was observed among a sample of male and female singletons born at very low birthweight (< 1,500 grams) followed from birth to 20 years (Hack et al., 2003). At birth, males had higher weight-for-age and height-for-age Z-scores relative to females, but by young adulthood females were indistinguishable from the population mean whereas males remained small (Hack et al., 2003). Previous research has found that females are less susceptible to neonatal complications compared to males, which may explain the faster rates of catch-up growth in females (Stevenson et al., 2000).

### Length Of Gestation And Physical Catch-up

Although gestational age emerged as an important environmental correlate of early weight, gestational age did not explain all of the variance in early physical size as birth weights and head circumference measurements for full-term infants were nearly 1SD below the population mean. Regarding birth length, full term infants were only slightly below the population mean (0.2 SD), whereas premature twins were nearly three quarters of a SD below the population mean. It is possible that factors associated with birth length (e.g., skeletal growth) benefit more from full gestation. However, measurements at birth are known to be unreliable (Laar et al., 2018) and may not have been performed on the unhealthiest neonates due to the medical risks associated with stretching a medically fragile newborn on a table to measure length (Hack et al., 2003). Indeed, rates of missing birth length were relatively high (20%) and there was a decline in length relative to the population mean between birth and 3 months when smaller infants could be safely measured (see Fig. 4).

Infants born earlier had a faster rate of height and weight catch-up growth and an earlier inflection point of height and head circumference growth, providing insight into the process by which children recover physically following premature birth. That is, premature infants caught up by growing more rapidly in early infancy than full-term infants (as opposed to more slowly but for a longer period of time). A variety of prenatal stressors associated with premature birth are also associated with restricted prenatal growth (e.g., preeclampsia, gestational diabetes, uterine size restrictions) (Goldenberg et al., 2008). Once prenatal environmental stressors are removed following birth, biological mechanisms may take over and stimulate physical growth.

### Nonlinear Associations Between Ses And Catch-up Growth

Accounting for gestational age, sex, and maternal age, children in wealthier families were born heavier and with larger heads, indicating more complete prenatal development. However, even children born in the highest-SES households displayed physical Deficits in early infancy. Children born in very wealthy and very poor homes had birth weights closest to the population mean at birth. Families at the highest end of the SES spectrum may have better access to high quality prenatal care and nutrition to support healthy prenatal development. Poverty may be related to higher birth weights through higher rates of gestational diabetes (Nagahawatte & Goldenberg, 2008), a prenatal complication associated with higher birth weights (Gillman et al., 2003). Alternatively, very impoverished families may be eligible to receive nutritional supplementation (e.g., Women, Infants, and Children supplemental nutrition), which has been linked to elevated birth weights (Kowaleski-Jones & Duncan, 2002).

Family SES was also quadratically related to the upper asymptote of height and weight. At the upper asymptote, children in the poorest homes weighted approximately 0.4 SD above the population mean and were approximately 0.2 SD taller than average. Family SES was unrelated to the rate of height or weight catch-up, indicating that children in the poorest homes grew relative to the population mean at the same rate as their average SES peers, but grew for a longer period of time. In the case of weight, the extended weight growth may highlight the developmental course of child- or adolescent-onset obesity among low birthweight children reared in poverty especially as upper asymptotes of weight were higher than upper asymptotes of height (Klebanov et al., 2014; Lee et al., 2014). The extended duration of weight gain may highlight the developmental course of adolescent-onset obesity among low-birth-weight children reared in poverty. Ongoing efforts to follow up with the Louisville Twins in middle adulthood (Beam et al., 2020) provide an important opportunity to extend our knowledge of genetic and environmental factors related to healthy and unhealthy weight gain across the lifespan.

### Limitations And Future Directions

Although there is socioeconomic variability in the LTS, it is an almost entirely White sample. Therefore, findings reflect the process of catch-up growth in White twins born in the United States and do not necessarily generalize to populations outside of the United States or nonwhite populations within the United States. For example, rates of malnutrition are extremely low in the United States (United Nations Children’s Fund, 2021) and social programs such as WIC centers and the Supplemental Nutrition Assistance Program allow even extremely impoverished families to meet the basic nutritional needs of their children (Kowaleski-Jones & Duncan, 2002). In developing countries where rates of malnutrition are higher and supplementary nutritional programs do not exist, shared environmental factors may explain a greater proportion of the variance in catch-up growth. Moreover, structural barriers that contribute to inequities in access to socioeconomic resources and medical care in the United States along racial lines (Bailey et al., 2017) may contribute to different patterns of physical development in Black, Latinx, and Indigenous populations. Additionally, experiences of racism and discrimination during pregnancy may elevate the risk for adverse prenatal outcomes and, therefore, represent an additional shared environmental experience that may contribute to individual differences in birth outcomes among Black, Latinx, and Indigenous populations (Sounderlund et al., 2021).

Rates of very premature birth and very low birth weight were low in this sample (~ 2%), and, therefore, findings reflect patterns of catch-up growth in a relatively low risk sample. Moreover, because the primary goal was to model typical trajectories of physical catch-up among twins, we did not distinguish small-for-gestational age twins from appropriate-for-gestational age twins. Examining physical developmental differences in small-for-gestational age versus appropriate-for-gestational age twins represents an important step for future research as children born small-for-gestational age status may experience greater prenatal stressors (McCowan & Horgan, 2009) and different patterns of postnatal development (Bertino et al., 2006).

We were limited to broad measures of the shared environment, which prohibited the exploration of more specific environmental correlates of physical catch-up. Gestational age was included as a crude indicator of prenatal health, and family SES at birth as a general indicator of the postnatal environment. Future work including more specific environmental experiences (e.g., prenatal exposure to heavy metals, postnatal diet) would provide further insight into environmental correlates of weight development in twins.

Finally, we are unable to make any causal claims regarding “effects” of genetics or environmental factors on catch-up growth in twins. Although the upper asymptotes of height, weight, and head circumference were highly heritable, it is not necessarily the case that one’s upper asymptote of physical size is caused by their genetics. Active or evocative gene-environment or phenotype-environment correlations may inflate the proportion of variance attributed to additive genetics (Beam & Turkheimer, 2013). For example, infant appetite, a highly heritable characteristic (Llewellyn et al., 2010), may contribute to one twin consuming more or requesting more frequent feeding. Genetic differences related to early appetite could contribute to within-pair differences in the rate or growth or upper asymptote of physical size for dizygotic twins.

## Conclusions

Catch-up growth in twins begins immediately after birth as evidence by the preferred fit of exponential-shaped functions over polynomial and S-shaped functions. Temporally, head circumference catch-up preceded height and weight catch-up. However, on average, substantial catch-up of all physical measures occurred within the first year of life. Consistent with previous research, gestational age was an important early environmental correlate of height, weight, and physical size. However, with the exception of birth length, twins born at full term continued to display substantial deficits in their physical size at birth. Therefore, additional prenatal factors associated with twinning (e.g., competition for nutrients) likely also contribute to the relatively small physical size of twins at birth. Importantly, there were no differences in childhood physical size between premature and full-term twins. Family SES also emerged as an important environmental correlate to physical catch-up growth in twins. Children in the wealthiest and poorest homes were closer to the population mean at birth in terms of weight and length. However, children in the poorest homes grew to be both heavier and taller than the population mean by adolescence. The relatively larger estimated weight among very poor children in adolescence (z-score = 0.4) compared to the estimated height (z-score = 0.2) suggests a possible like between poverty and later obesity among children born at low birth weight through extended weight gain across childhood.

Although the present study focused on a sample of twins, findings likely generalize to singletons who experience early bioenvironmental risk. As with twins, most singletons who are born at low birth weight, short birth length, or with a small head circumference at birth catch up to population norms (Lundgren et al., 2001), and substantial catch-up growth appears to occur across the first few months of life (Albertsson-Wikland & Karlberg, 1997). Pediatricians and primary care physicians working with children who are born prematurely or physically small should expect to see rapid catch-up growth in physical size across the first year. Children who are not making progress toward the population mean on age-standardized measurements over the first year may be at risk to remain physically small throughout their lives and may benefit from more rigorous intervention to encourage catch-up growth (e.g., nutritional supplementation).

## Figures and Tables

**Table I:** Parameter Estimates: Height Catch-Up Growth

Parameter	Mean [95% C.I.]	Within-Pair Variance MZ [95% C.I.]	Between-Pair Variance MZ [95% C.I.]	Within-Pair Variance DZ [95% C.I.]	Between-Pair Variance DZ [95% C.I.]
Intercept (*b*_*0*_)	**−0.66 [−0.75, −0.58]**	**0.13 [0.09, 0.16]**	**0.79 [0.68, 0.90]**	**0.26 [0.19, 0.32]**	**0.66 [0.53, 0.78]**
Upper Asymptote (*b*_*1*_)	**−0.11 [−0.18, −0.03]**	**0.05 [0.04, 0.06]**	**0.91 [0.81, 1.01]**	**0.44 [0.35, 0.53]**	**0.52 [0.40, 0.63]**
Rate of Growth (*b*_*2*_)	**0.31 [0.28, 0.34]**	**0.02 [0.01, 0.03]**	**0.17 [0.10, 0.24]**	**0.05 [0.03, 0.08]**	**0.13 [0.08, 0.19]**
Inflection Point Scaling Parameter (*b3*)	**1.60 [1.42, 1.78]**	**1.15 [0.52, 1.79]**	**9.17 [5.40, 12.93]**	**2.06 [0.83, 3.29]**	**8.27 [4.76, 11.78]**

	Biometric Contributions		
	Additive Genetic (A) [95% C.I.]	Shared Environment (C) [95% C.I.]	Nonshared Environment (E) [95% C.I.]
Intercept (*b*_*0*_)	**0.26 [0.11, 0.41]**	**0.53 [0.35, 0.71]**	**0.13 [0.09, 0.16]**
Upper Asymptote (*b*_*1*_)	**0.78 [0.60, 0.95]**	0.13 [−0.05, 0.31]	**0.05 [0.04, 0.06]**
Rate of Growth (*b*_*2*_)	**0.07 [0.03, 0.11]**	**0.10 [0.05, 0.15]**	**0.02 [0.01, 0.03]**
Inflection Point Scaling Parameter (*b3*)	1.80 [−0.31, 3.92]	**7.34 [3.80, 10.93]**	**0.20 [0.10, 0.30]**

*Note*. Estimates that are significantly different from 0 at *p* < .05 are presented in bold.

**Table II:** Correlates of Height Catch-Up

Predictor Variable	Intercept (*b*_*0*_) *B* [95% C.I.]	Upper Asymptote (*b*_*1*_) *B* [95% C.I.]	Rate of Change (*b*_*2*_) *B* [95% C.I.]	Inflection Point Scaler (*b*_*3*_) B*B* [95% C.I.]
Sex	**−0.21 [−0.36, −0.05]**	0.10 [−0.05, 0.25]	**−0.15 [−0.24, −0.06]**	**−0.85 [−1.61, −0.09]**
Family SES	0.02 [−0.07, 0.13]	−0.01 [−0.09, 0.07]	0.00 [−0.05, 0.05]	0.03 [−0.30, 0.37]
Family SES^2^	0.08 [−0.01, 0.17]	**0.10 [0.02, 0.18]**	−0.03 [−0.08, 0.02]	0.23 [−0.09, 0.56]
Gestational Age	**0.40 [0.27, 0.53]**	0.10 [−0.00, 0.20]	**−0.10 [−0.17, −0.03]**	**0.91 [0.52, 1.29]**
Maternal Age	**0.10 [0.02, 0.19]**	0.03 [−0.05, 0.11]	0.03 [−0.02, 0.08]	0.18 [−0.18, 0.54]

*Note*. Coefficients that are significant at the *p* < .05 level are highlighted in bold. Prior to centering, sex was coded such that males = 1 and females = 0.

**Table III:** Parameter Estimates: Weight Catch-Up Growth

Parameter	Mean [95% C.I.]	Within-Pair Variance MZ [95% C.I.]	Between-Pair Variance MZ [95% C.I.]	Within-Pair Variance DZ [95% C.I.]	Between-Pair Variance DZ [95% C.I.]
Intercept (*b*_*0*_)	**−1.58 [−1.64, −1.52]**	**0.14 [0.08, 0.21]**	**0.44 [0.37, 0.52]**	**0.17 [0.05, 0.30]**	**−1.58 [−1.64, −1.52]**
Upper Asymptote (*b*_*1*_)	**−0.13 [−0.20, −0.05]**	**0.09 [0.07, 0.11]**	**1.00 [0.85, 1.14]**	**0.49 [0.37, 0.61]**	**−0.13 [−0.20, −0.05]**
Rate of Growth (*b*_*2*_)	**0.81 [0.72, 0.90]**	**0.13 [0.06, 0.21]**	**2.18 [1.20, 3.16]**	**0.91 [0.48, 1.33]**	**0.81 [0.72, 0.90]**
Inflection Point Scaling Parameter (*b3*)	**0.80 [0.73, 0.86]**	**0.08 [0.04, 0.11]**	**1.01 [0.77, 1.26]**	**0.37 [0.22, 0.51]**	**0.80 [0.73, 0.86]**

	Biometric Contributions		
	Additive Genetic (A) [95% C.I.]	Shared Environment (C) [95% C.I.]	Nonshared Environment (E) [95% C.I.]
Intercept (*b*_*0*_)	0.06 [−0.21, 0.34]	**0.38 [0.11, 0.66]**	**0.14 [0.08, 0.21]**
Upper Asymptote (*b*_*1*_)	**0.80 [0.55, 1.04]**	0.20 [−0.03, 0.43]	**0.09 [0.07, 0.11]**
Rate of Growth (*b*_*2*_)	**1.55 [0.78, 2.32]**	**0.63 [0.02, 1.23]**	**0.13 [0.06, 0.21]**
Inflection Point Scaling Parameter (*b3*)	**0.58 [0.29, 0.87]**	**0.43 [0.12, 0.74]**	**0.08 [0.04, 0.11]**

*Note*. Estimates that are significantly different from 0 at *p* < .05 are presented in bold.

**Table IV:** Correlates of Weight Catch-Up

Between-Pair Effects				
Predictor Variable	Intercept (*b*_*0*_) *B* [95% C.I.]	Upper Asymptote (*b*_*1*_) *B* [95% C.I.]	Rate of Change (*b*_*2*_) *B* [95% C.I.]	Inflection Point (*b*_*3*_) *B* [95% C.I.]
Sex	**0.16 [0.07, 0.25]**	−0.00 [−0.17, 0.16]	**−0.33 [−0.60, −0.05]**	**0.30 [0.10, 0.50]**
Family SES	0.03 [−0.02, 0.09]	−0.10 [−0.19, 0.00]	0.07 [−0.08, 0.22]	0.04 [−0.06, 0.14]
Family SES^2^*	**0.08 [0.02, 0.13]**	**0.11 [0.01, 0.20]**	−0.04 [−0.20, 0.11]	−0.04 [−0.15, 0.08]
Gestational Age	**0.49 [0.44, 0.53]**	**0.10 [0.00, 0.18]**	**−0.17 [−0.31, −0.04]**	−0.01 [−0.13, 0.11]
Maternal Age	0.04 [−0.01, 0.10]	−0.01 [−0.10, 0.07]	0.13 [−0.05, 0.26]	**0.13 [0.03, 0.23]**

*Note*. Coefficients that are significant at the *p* < .05 level are highlighted in bold.

* Family SES^2^ is the residual of family SES squared regressed onto family SES.

**Table V:** Parameter Estimates: Head Circumference Catch-Up Growth

Parameter	Mean [95% C.I.]	Within-Pair Variance MZ [95% C.I.]	Between-Pair Variance MZ [95% C.I.]	Within-Pair Variance DZ [95% C.I.]	Between-Pair Variance DZ [95% C.I.]
Intercept (*b*_*0*_)	**−1.47 [−1.59, −1.36]**	**0.22 [0.16, 0.28]**	**0.70 [0.55, 0.86]**	**0.30 [0.22, 0.38]**	**0.63 [0.47, 0.79]**
Upper Asymptote (*b*_*1*_)	**0.46 [0.38, 0.53]**	**0.05 [0.04, 0.07]**	**0.76 [0.66, 0.86]**	**0.43 [0.33, 0.54]**	**0.38 [0.27, 0.50]**
Rate of Growth (*b*_*2*_)	**2.28 [2.08, 2.48]**	**0.31 [0.15, 0.48]**	**1.45 [0.83, 2.06]**	**0.92 [0.55, 1.30]**	**0.84 [0.27, 1.41]**
Inflection Point Scaling Parameter (*b3*)	**1.97 [1.79, 2.14]**	**0.67 [0.34, 1.00]**	**1.25 [0.77, 1.73]**	**0.67 [0.34, 1.00]**	**1.25 [0.77, 1.73]**

	Biometric Contributions		
	Additive Genetic (A) [95% C.I.]	Shared Environment (C) [95% C.I.]	Nonshared Environment (E) [95% C.I.]
Intercept (*b*_*0*_)	**0.63 [0.47, 0.79]**	0.15 [−0.04, 0.34]	**0.55 [0.34, 0.76]**
Upper Asymptote (*b*_*1*_)	**0.38 [0.27, 0.50]**	**0.75 [0.54, 0.97]**	0.01 [−0.20, 0.21]
Rate of Growth (*b*_*2*_)	**0.84 [0.27, 1.41]**	**1.22 [0.53, 1.91]**	0.23 [−0.49, 0.94]
Inflection Point Scaling Parameter (*b3*)	**1.25 [0.77, 1.73]**	0*	**1.25 [0.77,1.73]**

*Note*. Estimates that are significantly different from 0 at *p* < .05 are presented in bold.

* The additive genetic variance for *b*_*3*_ was constrained to 0 as it was initially estimated to be negative and non-significantly different than 0.

**Table 6:** Correlates of Head Circumference Catch-Up

Predictor Variable	Intercept (*b*_*0*_) *B* [95% C.I.]	Upper Asymptote (*b*_*1*_) *B* [95% C.I.]	Rate of Change (*b*_*2*_) *B* [95% C.I.]	Inflection Point (*b*_*3*_) *B* [95% C.I.]
Sex	**0.30 [0.15, 0.44]**	0.10 [−0.04, 0.24]	**−0.69 [−0.97, −0.42]**	**0.14 [0.15, 0.42]**
Family SES	**0.12 [0.04, 0.21]**	**0.09 [0.01, 0.17]**	−0.09 [−0.23, 0.06]	0.09 [−0.06, 0.24]
Family SES^2^*	0.02 [−0.06, 0.10]	0.03 [−0.04, 0.10]	0.00 [−0.12, 0.13]	−0.02 [−0.17, 0.12]
Gestational Age	**0.57 [0.49, 0.66]**	−0.03 [−0.11, 0.06]	−0.12 [−0.27, 0.02]	**0.17 [0.02, 0.33]**
Maternal Age	0.02 [−0.08, 0.11]	0.03 [−0.04, 0.10]	0.05 [−0.09, 0.20]	0.02 [−0.14, 0.18]

*Note*. Coefficients that are significant at the *p* < .05 level are highlighted in bold.

* Family SES^2^ is the residual of family SES squared regressed onto family SES.

## Data Availability

The data that support the findings of this study are not publicly available. Data may be made available upon reasonable request from the principal investigators, [CB and DD], upon reasonable request.
